# Strategies for the Long-Term Preservation of Site for Future Implantation of Cardiac Implantable Electronic Devices (CIEDs): Two Decades of Experience

**DOI:** 10.7759/cureus.22259

**Published:** 2022-02-15

**Authors:** Bakhtawar Shah, Shahab Saidullah, Muhammad Aamer Niaz, Farrukh Zaman, Zahida Parveen, Aamir Ghazanfar, Hassan Mumtaz

**Affiliations:** 1 Clinical Cardiac Electrophysiology, Hayatabad Medical Complex Peshawar, Peshawar, PAK; 2 Cardiology, Pakistan Institute of Medical Sciences, Islamabad, PAK; 3 Interventional Cardiology, Hayatabad Medical Complex Peshawar, Peshawar, PAK; 4 Diabetes and Endocrinology, Kahuta Research Laboratory (KRL) Hospital, Islamabad, PAK; 5 Gynecology, Kahuta Research Laboratory (KRL) Hospital, Islamabad, PAK; 6 General Surgery, Kahuta Research Laboratory (KRL) Hospital, Islamabad, PAK; 7 General Medicine, Surrey Docks Health Center, London, GBR; 8 Public Health, Health Services Academy, Islamabad, PAK; 9 Clinical Research, Maroof International Hospital, Islamabad, PAK; 10 Urology, Guy's & St Thomas' NHS Foundation Trust, London, GBR

**Keywords:** insulation break, lead fracture, right ventricle perforation, lead displacement, lead & device erosion

## Abstract

Introduction

Implantation of cardiac implantable electronic devices (CIEDs) is an art of science. As the volume of implantation has increased worldwide, so has the rate of complications. Infection, fibrosis, lead and device erosion, lead displacement, right ventricle perforation, lead fracture, and insulation break are the common complications in the implantation process. This exposes the patient for reopening and threatens the implantation for further complication due to infection, fibrosis of veins, failure to retrieve the implanted wire, and failure to re-implant the device on the same site. We slightly changed our implantation technique to preserve the implantation site for future implantation and reduce the rate of complication in the index implantation.

Methods

This randomized control trial was conducted from January 2016 to September 2019 at Hayatabad Medical Complex Peshawar, Pakistan. A consecutive sampling technique was used to obtain a sample size of 602 patients keeping a 95% confidence interval and a 5% margin error. We adopted a strategy to take prick, for implantation of devices, inside the pocket, which reduces the number of sutures, hastens the procedure, prevents erosion, and minimizes the chance of subclavian crush syndrome and insulation break. We also selected the minimum possible length of leads. This will possibly decrease the chances of cumbersome fibrosis around the lead and device and will make future implantation convenient.

Results

There was a total of 602 procedures in the study period. About 253 (42%) procedures were done in the newly adopted strategy and 349 (58%) were performed in the conventional way. Our complication rate grossly reduces in the novel way of implantation in which we took our prick inside the pocket.

Conclusion

A slight modification in the implantation of CIEDs not only prevents the rate of complication in the index implantation but will also possibly preserve the site for future implantation.

## Introduction

Implantation of cardiac implantable electronic devices (CIEDs) has become an integral part of cardiology in general and of electrophysiology in particular [1]. Implantation of cardiac devices is not a one-time procedure. Most cardiac devices remain functional from 8-10 years after which the pulse generator needs to be replaced. People who become dependent on devices at a very early age need multiple implantations in their lifetime. Mostly the same leads from the previous implantation are used and only the device is replaced. Therefore, explantation needs to be carefully handled and the device and leads freed from adhesion and fibrosis to successfully implant the new device.

Cardiac device implantations are very prone to complications [2]. These complications range from very mild discomfort to serious consequences, which can put at risk the life of the patient. The most common complication with cardiac devices are pocket hematoma, erosion, lead displacement, insulation break, lead fracture, subclavian crush syndrome, right-sided cardiac chambers perforation, and endocarditis associated with fibrosis, infection, and lead [3]. Multiple implantations not only increase the rate of complication but also make future implantation difficult at the same site [4].

CIEDs implantation needs special care to make the index implantation successful, preserve the site for future implantation and replacement, and possibly reduce the rate of potential future complications [5]. These precautions start in the preoperative period when the device implantation is planned and stay throughout the postoperative period for the rest of the patient’s life. The dreaded complications are implantation site infection, fibrosis around the lead and devices, stenosis of the veins, and erosion of lead and devices. All these complications will make future implantation cumbersome [2].

Our big enemies in the implantation of CIEDs, in developing counties, are infections [6]. This not only spoils the index implantation but makes future implantation difficult and often endangers the life of the patient. It may be due to faulty implantation surgical skill, erosion of the devices, or trauma to the device’s site [7]. The other common complication that makes future implantation difficult or impossible is fibrosis around the device and inside the vein [8].

Preserving the implantation site is the key for future implantation. During our implantation of CIEDs, we experienced almost all the mentioned complications in the past two decades and, therefore, we continuously reviewed our implantation procedure, not only to minimize the rate of complication but to preserve the site for future implantation and make the re-implantation easy. In this article we share our own experience in the field of implantation and preservation of implantation sites.

## Materials and methods

This randomized control trial was conducted from January 2016 to September 2019 at Hayatabad Medical Complex Peshawar, Pakistan. Using the reference sample size from a study published in 2018 [9] and the WHO calculator, a sample size of 602 patients was calculated with a 95% confidence interval and a 5% margin error. A consecutive sampling technique was used. Patients were randomly divided into two groups. Group “A” patients received CIEDs implantation in the conventional way and group “B” patients received the implantation of devices in the new way we adopted for device implantation.

The study was approved by the Scientific Review Committee and Ethical Committee (Human Research) of Post Graduate Medical Institute (PGMI), Peshawar, Pakistan.

Inclusion and exclusion criteria

All patients, irrespective of age, who presented for device implantation as elective cases or as emergency cases were included in our study. Patients who presented with device or lead erosion and patients who were having infection at the site of implantation were excluded from our study. 

Data collection

All those patients who presented to our unit for an elective procedure of implantation of CIEDs were admitted a day before the procedure. Patients who presented to emergency room and were in need of temporary pacemakers, or some other emergency procedure, got admitted at the time of presentation.

Informed consent was obtained. Baseline investigations including renal function test (RFT), serum electrolytes, random blood sugar (RBS), virology, full blood count (FBC), liver function test (LFT), coagulation profile, and cardiac enzyme blood test were advised according to the hospital protocol. Night bath was offered to those who were able to do so. Male patients’ chests were shaved at night. Both male and female patient chests were painted with Pyodine. Night sedation with 3 mg bromazepam orally administered.

Patients were brought nil by mouth to the catheterization laboratory. Intravenous (IV) cannula were passed on both upper limbs. Premedication with IV dimenhydrinate, injection nalbuphine, and at time, injection midazolam was given. It is our usual practice to give IV antibiotics half an hour before surgery, and second dose during surgery or just at the end of procedure if implantation time is less 30 minutes.

After patient was scrubbed, draped venography was performed of the axillary vein on the side of implantation and local anesthesia with 2% lidocaine was infiltrated.

Group “A” patients: Devicewas implanted in the conventional way of implantation. After skin incision and pocket construction, guide wire was passed through Seldinger technique and sheath passed. After removal of wire and dilator, lead was implanted, battery attached, and pocket was closed. Lead berried in muscle and wound closed in layers.

Group “B” patients: Through the intact skin, axillary vein puncture was done by Seldinger technique and guide wire passed. About 2-3 cm superolateral to the puncture site, skin incision was made and self-retaining retractor applied. With blunt dissection, the subcutaneous tissues were separated using artery forceps till the deep fascia over the muscle was visible. Electric cattery was used to keep the field clean and dry. With tooth forceps, the flap of the skin is left and dissection extended infero-medially to construct the pocket for device. The preplaced guide wires were identified.

Once the guide wire came into the site, they were pulled in from inside the pocket, so that the outer end came in and out through the incision while the venous end was in place. In this way the puncture site goes inside the pocket. Now the sheath was passed over the wire and wire pulled out with dilator and PPM lead passed and secured. The lead was stabilized with muscle using 1/0 non-absorbable sutures. The pocket for pulse generator was irrigated with gentamicin so to remove any residual blood clots.

The device was attached with pacemakers leads, and battery pushed inside the pocket and stabilized with 1/0 silk inside the pocket. The pocket mouth was closed with 1/0 Vicryl and the skin was closed with interrupted non-absorbable sutures. Pressure dressing was applied for 24 hours to prevent any possible pocket hematoma. Patients were kept on IV antibiotics for five days according to our hospital microbiology protocol. Patients were discharged on oral antibiotics for five more days and skin stitches were removed on day 15 of procedure.

Data analysis

IBM SPSS Statistics for Windows, Version 22.0 (Released 2013, IBM Corp., Armonk, New York, United States) was used for statistical analysis involving quantitative variables.

## Results

**Figure 1:**
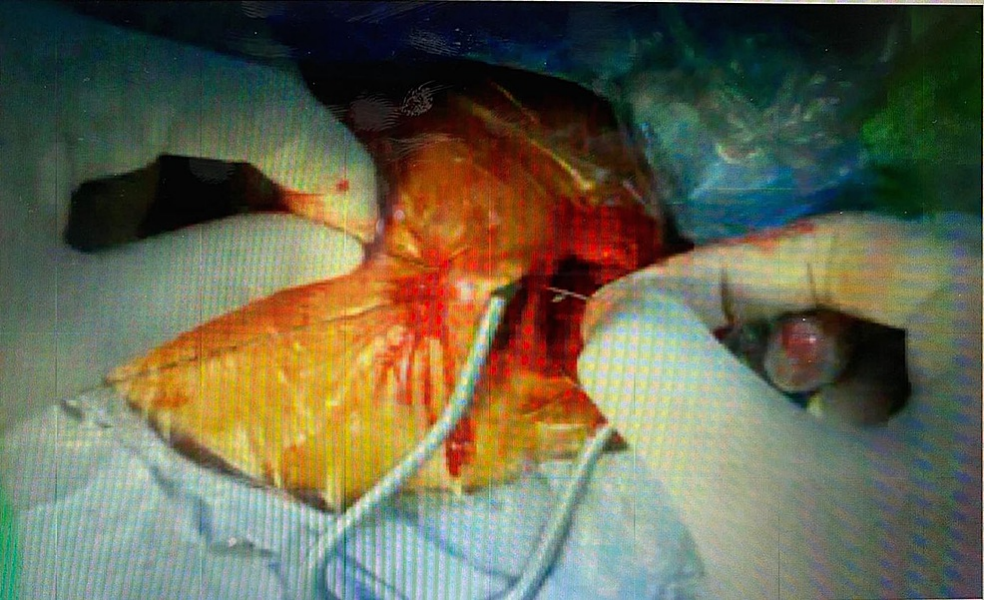
Demonstration of prick site and incision line

There were a total of 602 patients in the study. The patients whose procedure was performed in the conventional way numbered 349 (58%). The new strategy was tried in 253 (42%) patients. The demographic data of the patients are tabulated in Table 1. There were 325 (54%) male patients and 277 (46%) female patients. However, there was no significant difference in the rate of complication in both genders. The age distribution was from 10 years to 100 years. The mean age of implantation remains 60.26±16.88 years. Lead displacement and lead damage were seen more frequently in the young age group while pneumothorax complicated the procedure above 60 years of age. The prick site and incision line is demonstrated in Figure 1.

**Table 1:** Baseline characteristic of patients VVI/VVIR: ventricular demand pacing single chamber pacemaker; DDD/DDDR: dual chamber rate adaptive pacemaker

DEMOGRAPHIC CHARACTERS	NUMBERS
Total Patients	602
Male	325 (54%)
Female	277 (46%)
Age	10 to 100 (60.26±16.88)years
Tine Lead	27 (4.5%)
Screwing Lead	573 (95.2%)
Tine And Screwing Leads	2 (0.3%)
VVI/VVIR	432 (71.8%)
DDD/DDDR	170 (28.2%)
New Implantation	550 (91.4%)
Box Change	19 (3.2%)
Upgradation Of Devices	1 (0.2%)
Reposition Of Lead	21 (3%)
Lead Abandoned	1 (0.2%)
Explantation	8 (1.3%)
Conventional Implantation Method	349 (58%)
Novel /Change Strategy	253 (42%)

The rate of pneumothorax was high in the conventional method of implantation, which is statistically significant. The comparison of complications in both our conventional method and novel changed strategy is tabulated in Table 2. The rate of almost all complications is more with dual-chamber pacemakers. These complications disturb the field of implantation and make future implantation cumbersome. However, single chambers devices have a very limited indication. 

**Table 2:** Complications in procedure

COMPLICATION IN PROCEDURE	METHOD	
	CONVENTIONAL	NOVEL
Lead Displacement	3	2
Mild Pericardial Effusion	0	1
Haematoma	2	0
Infection	1	0
Pneumothorax	7	2
Lead Damage	1	1

Tine leads are almost obsolete in today's practice, but we had 27 tine leads in our study population. There were 21 new implantations of tine leads and mostly they were implanted in atria, which were pre-shaped leads. So there is no chance of displacement in these leads. However, the retrieval rate is significantly low with these leads, and most of the time they are abandoned if there is damage to the leads.

## Discussion

As CIEDs implantation is not a one-time procedure, re-implantation of CIEDs is a cumbersome procedure [10]. Re-implantation of the device by itself is not only difficult but it is much more prone to infection as compared to the primary procedure [11]. The recovery of the previous CIED and re-implantation success mostly depends on the first procedure. If the anatomy of the implantation site is destroyed at the time of primary implantation, the subsequent procedure will be very much difficult. Therefore preservation of the implantation site is of utmost importance. If the first procedure is performed in a way that there is hematoma formation, infection, and a lot of adhesion, then the repeat procedure will be not only difficult but may lead to failure of successful implantation [12].

Small hematoma and serous collection are common after implantation of CIEDs [13]. But large hematoma at the implantation site makes the site prone to dehisces, infection, adhesion, and delayed healing [14]. Apart from this, it will make the area unsuitable for subsequent procedures. Therefore, cautious hematoma prevention at first implantation will not only prevent failure of the first procedure but will make the following procedures a success. This will decrease the ratio of adhesion and the retrieval of the first lead and device will be easy.

Repeated unsuccessful attempts to prick the vein, and consequently hitting the axillary artery repeatedly, increase the chance of hematoma formation [15]. Therefore prior identification of the vein site by a venogram or ultrasound [16] and avoiding incidental pricking of the axillary artery will prevent hematoma. Plugging and securing the lead sleeve properly at the entry point in muscle also prevents oozing and collection of hematoma [17].

If the lead is lying directly in the line of incision, then it is necessary to bury it in muscles to avoid lead erosion, particularly in very young and elderly populations [2]. But this will increase the chance of adhesion in the future and retrieval of lead will be difficult. Similarly, the number of leads also affects the outcome. An increased number of leads will complicate the procedure in several ways. It increases the rate of procedure-related complications and also the increased number of hardware leads to increased adhesions and fibrosis [18]. But multiple leads are almost always needed in different morbidities and indication for a single chamber is very limited. Therefore, we adopted a technique to accommodate the whole hardware in the pocket in a way that it is not only in a well-protected position but also minimizes the procedure-related complication and saves the area for future implantation.

Increased numbers of suture material also increase the chance of infection and adhesion [19]. The suture martial was much reduced in our new technique. Only one suture is sufficient to bury the whole hardware in the pocket and there is less chance of infection and lead erosion. The chance of wound dehiscence due to pressure on the suture line is negligible.

The increased length of the lead and putting a lot of lead folded under the device also increase adhesion and subsequent retrieval difficulty [18]. Therefore, we selected the minimum possible length of the lead, which prevented the over folding of lead and decreased the cumbersome adhesions. This makes the retrieval easy subsequently and also prevents damage to the area for subsequent implantation.

Electrocautery reduces the chance of hematoma and keeps the implantation site dry [20]. Similarly, irrigating the pocket with saline and antibiotics to wash out any clotted blood also reduces the chance of infection and adhesions [21,22]. Applying pressure bandage to the wound site is very helpful in reducing collection and makes the recovery speedy [23]. Therefore, there is less chance of subsequent adhesion.

We do not practice the cut-down procedure of the cephalic vein [24] anymore, because there is more manipulation of structure, which ultimately increases fibrosis and makes future implantation more difficult. Skin incision at the time of explantation of the device is very important. Most operators cut the skin over the device, which will make the access easy to the previously implanted device but re-implantation becomes difficult at the same time and the pocket is needed to be extended downward to accommodate the hardware in the pocket. This process makes the procedure prone to further complications. Therefore, we give an incision at or near the old incision and carefully dissect the area to retrieve the device. This technique increases the time of explantation and re-implantation but preserves the same implantation site for future repeated implantation.

As infection is our biggest enemy, which not only spoils the index procedure but also leads to the destruction of the site for future implantation [25], we offer a night bath before the procedure if possible, paint the site with Pyodine solution at night, and give inject able broad-spectrum antibiotics an hour before the procedure and the second dose during the procedure. Patients remain on antibiotics for ten days to prevent infection. Patients with temporary pacemakers (TPM) are started on antibiotics at the time of implantation of TPM and remain on antibiotics till the patients get a permanent pacemaker (PPM).

No matter how careful and equipped we are, still, some unavoidable conditions pop up in the process of CIEDs implantation. Abnormal response of the human body to foreign materials is a cumbersome complication [26,27], which leads to exaggerated fibrosis and stenosis of veins. Leadless pacemakers [28] are best for the prevention of subclavian area and vein stenosis but it does not fulfill all the aims of CIEDs implantation. Future research of stem cell therapy will possibly be the right answer to this question.

Limitation of the study

Results are in favor of our change strategy as far as the procedure-related complications and efficacy of the procedure are concerned but we need time to compare the re-exploration results of the two approaches.

## Conclusions

As CIEDs implantation is not a one-time procedure, therefore, the site of implantation needs to be preserved. If the rate of complication could be reduced, the area of implantation may be preserved for a long period of time. The complications rate is less with axillary vein prick and using an appropriate length of leads. Carefully positioning the lead at the time of implantation, so as to avoid displacement and repositioning will reduce complications. Last but not least, minimizing the chance of infection by appropriate use of antibiotics is the key to preventing erosion and preserving the site.

## References

[1] Sood N, Martin DT, Lampert R, Curtis JP, Parzynski C, Clancy J (2018). Incidence and predictors of perioperative complications with transvenous lead extractions: real-world experience with national cardiovascular data registry. Circ Arrhythm Electrophysiol.

[2] Garg N, Moorthy N (2012). Pacemaker lead erosion simulating "Loch Ness Monster": conservative management. Asian Cardiovasc Thorac Ann.

[3] Tjong FV, Reddy VY (2017). Permanent leadless cardiac pacemaker therapy: a comprehensive review. Circulation.

[4] Ganesan AN, Moore K, Horton D (2020). Complications of cardiac implantable electronic device placement in public and private hospitals. Intern Med J.

[5] Love CJ (2018). Lead management and lead extraction. Card Electrophysiol Clin.

[6] Alp E, Elmali F, Ersoy S, Kucuk C, Doganay M (2014). Incidence and risk factors of surgical site infection in general surgery in a developing country. Surg Today.

[7] VanEpps JS, Younger JG (2016). Implantable device-related infection. Shock.

[8] Burri H (2015). Overcoming the challenge of venous occlusion for lead implantation. Indian Pacing Electrophysiol J.

[9] Raja TA, Kashif M, Rehman AU, Kiyani A, Shabbir F, Rajput TA (2018). Indication and frequency of implantation of permanent pacemaker in complete heart block patients in a tertiary care hospital in Rawalpindi. PAFMJ.

[10] Smith MC, Love CJ (2008). Extraction of transvenous pacing and ICD leads. Pacing Clin Electrophysiol.

[11] Chew D, Somayaji R, Conly J, Exner D, Rennert-May E (2019). Timing of device reimplantation and reinfection rates following cardiac implantable electronic device infection: a systematic review and meta-analysis. BMJ Open.

[12] Boyle TA, Uslan DZ, Prutkin JM (2017). Reimplantation and repeat infection after cardiac-implantable electronic device infections: experience from the MEDIC (multicenter electrophysiologic device infection cohort) database. Circ Arrhythm Electrophysiol.

[13] Thal S, Moukabary T, Boyella R, Shanmugasundaram M, Pierce MK, Thai H, Goldman S (2010). The relationship between warfarin, aspirin, and clopidogrel continuation in the peri-procedural period and the incidence of hematoma formation after device implantation. Pacing Clin Electrophysiol.

[14] Gabriel A, Gupta S, Orgill DP (2019). Challenges and management of surgical site occurrences. Plast Reconstr Surg.

[15] Tan K, Tan LW, Chia PL, Foo D (2019). A case of unstable occult arterial bleeding post pacemaker implantation. J Arrhythm.

[16] Seto AH, Jolly A, Salcedo J (2013). Ultrasound-guided venous access for pacemakers and defibrillators. J Cardiovasc Electrophysiol.

[17] Rezazadeh S, Wang S, Rizkallah J (2019). Evaluation of common suturing techniques to secure implantable cardiac electronic device leads: Which strategy best reduces the lead dislodgement risk?. Can J Surg.

[18] Gould J, Sidhu BS, Porter B (2019). Prolonged lead dwell time and lead burden predict bailout transfemoral lead extraction. Pacing Clin Electrophysiol.

[19] Mahesh L, Kumar VR, Jain A (2019). Bacterial adherence around sutures of different material at grafted site: a microbiological analysis. Materials (Basel).

[20] Wiegand UK, LeJeune D, Boguschewski F, Bonnemeier H, Eberhardt F, Schunkert H, Bode F (2004). Pocket hematoma after pacemaker or implantable cardioverter defibrillator surgery: influence of patient morbidity, operation strategy, and perioperative antiplatelet/anticoagulation therapy. Chest.

[21] Kang FG (2019). Confounding factors on the prediction of opioid usage after thyroidectomy and parathyroidectomy surgery. Otolaryngol Head Neck Surg.

[22] Lakshmanadoss U, Nuanez B, Kutinsky I, Khalid R, Haines DE, Wong WS (2016). Incidence of pocket infection postcardiac device implantation using antibiotic versus saline solution for pocket irrigation. Pacing Clin Electrophysiol.

[23] Chien CY, Chang YH, Wu YJ (2019). Effectiveness of a non-taped compression dress in patients receiving cardiac implantable electronic devices. Acta Cardiol Sin.

[24] Kolettis TM, Lysitsas DN, Apostolidis D, Baltogiannis GG, Sourla E, Michalis LK (2010). Improved 'cut-down' technique for transvenous pacemaker lead implantation. Europace.

[25] Al-Maisary SS, Romano G, Karck M, De Simone R (2019). Epicardial pacemaker as a bridge for pacemaker-dependent patients undergoing explantation of infected cardiac implantable electronic devices. J Card Surg.

[26] Shittu M, Shah P, Elkhalili W, Suleiman A, Shaaban H, Shah PA, Shamoon F (2015). A rare case of recurrent pacemaker allergic reaction. Heart Views.

[27] Abdallah HI, Balsara RK, O'Riordan AC (1994). Pacemaker contact sensitivity: clinical recognition and management. Ann Thorac Surg.

[28] El-Chami MF, Merchant FM, Leon AR (2017). Leadless pacemakers. Am J Cardiol.

